# Association between COVID-19 infection experience and physical activity levels in post-recovery college students

**DOI:** 10.3389/fpubh.2026.1756698

**Published:** 2026-07-03

**Authors:** Zhiyang Guan, Yiting Wu, Gaojie Liu, Maolin You

**Affiliations:** 1 School of Physical Education, Jiangxi Normal University, Jiangxi Normal University, Nanchang, China; 2 School of Physical Education, School of Soochow University, Suzhou, China

**Keywords:** anti-pandemic exercise, college students, level of physical activity, national fitness, virus infection experience

## Abstract

**Objective:**

This study aimed to assess the level of physical activity in college students after recovery from coronavirus disease 2019 (COVID-19).

**Methods:**

The Physical Activity Rating Scale (PARS-3) and the Wisconsin Upper Respiratory Symptom Survey (WURSS-21) questionnaire were used to conduct a survey on 1,633 college students.

**Results:**

(1) The experience of COVID-19 was negatively correlated with the level of physical activity after recovery (*r* = −0.230, *p* < 0.01). However, this effect size is small and statistical significance may be influenced by the large sample size. (2) The intensity and duration of physical activity showed the following canonical variates: U1 = −0.438X₁ (nasal symptoms) + 0.299X₂ (pharyngeal symptoms) + 1.144X₃ (activity/function) and V1 = −1.073Y₁ (intensity of physical activity) + 0.599Y₂ (duration of physical activity). The canonical correlation between U1 and V1 was 0.118 (*p* < 0.001). (3) Among male college students, the experience of COVID-19 infection was significantly negatively correlated with the overall level of physical activity (*r* = −0.188, *p* < 0.01). Specifically, the Spearman correlation coefficients revealed significant negative correlations with the intensity (*r* = −0.129, *p* < 0.01), duration (*r* = −0.080, *p* < 0.05), and frequency (*r* = −0.106, *p* < 0.01) of physical activity. In contrast, for female college students, the infection experience showed a non-significant negative correlation with the level of physical activity (*r* = −0.045, *p* > 0.05). The Spearman correlation coefficients for female students indicated a significant negative correlation with the intensity of physical activity (*r* = −0.081, small effect size, *p* < 0.05), while the correlations with duration (*r* = 0.061, *p* > 0.05) and frequency (*r* = 0.076, *p* > 0.05) were not significant. (4) The level of physical activity was lower with increased severity of COVID-19 experience. The overall decrease was 3.20 points for each level of increase in severity, with the individual decreases in male and female students being 5.98 and 2.79 points, respectively. Given the small effect sizes (*R*^2^ = 0.015–0.035), these differences, while statistically significant, may have limited practical significance.

**Conclusion:**

The experience of COVID-19 was associated with lower levels of physical activity among Chinese college students in the post-recovery phase, with a stronger negative correlation observed in male students. Notably, the duration and frequency of physical activity were higher among female students after recovery from COVID-19. These results are consistent with the importance of physical activity awareness during a pandemic, though causal inferences are precluded by the cross-sectional design. Additionally, the development of efficient fitness programs may be considered to support participation and address post-pandemic health needs.

## 1 Introduction

Pandemic outbreaks are associated with profound changes in various aspects of our lives, often coinciding with significant disruptions (1). For instance, Dr. Ide’s extensive 15-year study of Ireland, which was severely affected by the 1918 Spanish flu pandemic, revealed that communities, places, and all aspects of life showed significant changes (2). In a related study, Lau et al. (3) observed higher levels of healthy behaviors among individuals, such as improved dietary habits and weight management, coinciding with the SARS outbreak in 2003. Furthermore, Karlberg et al. (4) identified a significant gender disparity in the mortality rate of SARS, with men experiencing a mortality rate of 21.9% compared to 13.2% in women (*p* < 0.0001). Given these historical findings, it is pertinent to inquire whether a similar gender difference persists in the context of the novel coronavirus (COVID-19) more than a decade later.

Lazarus (5) proposed that individuals constantly evaluate the importance of external stimuli and adjust their behavioral responses, which may be associated with changes in their daily behaviors. For instance, individuals who have experienced myocardial infarction (MI) may become more aware of the risks associated with their previous habits and lifestyles, a period that coincided with higher levels of physical activity after being discharged from the hospital (6). Similarly, individuals with diabetes reported higher levels of physical activity, such as the frequency of walks (7). Therefore, the experience of illness and its co-occurrence with health changes may be associated with changes in perceptions and behaviors of individuals, potentially coinciding with the adoption of positive measures after recovery, such as engaging in physical activity. This phenomenon has been observed during various disease outbreaks, such as the severe acute respiratory syndrome (SARS) epidemic and the coronavirus disease 2019 (COVID-19) pandemic, wherein individuals reported higher physical activity levels coinciding with the outbreaks (8, 9). In a survey conducted by the Cleveland Clinic (10), approximately two-thirds of respondents reported major lifestyle changes, including the initiation or modification of physical activity routines, after the COVID-19 outbreak. However, lower physical activity levels were reported by some individuals following the pandemic subsidence (11). More than 90% of the population was infected with COVID-19 in China by December 7, 2022, and several government policies were implemented to control the outbreak, and many individuals shared their infection experiences on social media platforms. It is important for us to understand how these experiences are associated with health life behaviors among the general population, such as physical activities. To date, no studies have comprehensively investigated the level of physical activity in individuals recovering from viral infections. In this study, we examined the association between COVID-19 infection experience and the level of physical activity in college students in the post-recovery phase. The findings may inform healthcare strategies related to physical fitness after a disease outbreak, though causal efficacy cannot be established.

## 2 Objects and methods

### 2.1 Study design

In May 2023, our research group utilized the Questionnaire Star platform^1^ to gather detailed data on the symptoms experienced by college students during COVID-19 infection and to assess their physical activity levels during the six-month period following recovery. The six-month recall period was selected to capture sustained post-acute physical activity patterns while minimizing recall decay associated with longer retrospective intervals. However, we acknowledge that retrospective self-report of physical activity over 6 months is susceptible to recall bias, including telescoping (misdating events) and selective memory for salient episodes. Participants may also have difficulty accurately recalling intensity and frequency of activities across extended periods. To ensure data quality and prevent duplicate submissions, the platform automatically implemented IP address monitoring to flag multiple responses from the same IP, and we additionally screened the raw data for suspicious response patterns (e.g., abnormally short completion intervals and identical answer sequences) during the data cleaning process. Physical education teachers from Nanchang University, Jiangxi Normal University, East China Jiaotong University, Jiangxi University of Technology, and Jiangxi Science & Technology Normal University (all located in Nanchang) were invited to assist in distributing the questionnaire to university students. A total of 1,698 students voluntarily and anonymously completed the questionnaire, and the responses were carefully screened to ensure the validity of the data. The inclusion criteria were as follows: (1) a confirmed history of COVID-19 infection as indicated by a total score of >0 on the Wisconsin Upper Respiratory Symptom Survey (WURSS-21) scale; (2) university students; and (3) completion of the questionnaire. The exclusion criteria were as follows: (1) missing responses and (2) no history of COVID-19. Based on these criteria, 12 and 53 participants were excluded owing to missing responses and the lack of experience of COVID-19, respectively. Finally, 1,633 valid questionnaires were obtained, resulting in an effective recovery rate of 96.17%.

### 2.2 Participants

Among these participants, 47.5% (775) were male students and 52.5% (858) were female students. In terms of the academic year, 26.8% (438), 46.4% (757), 16.1% (263), and 10.7% (175) of the participants were freshmen, sopho-mores, juniors, and seniors, respectively. The average age of the participants was 19.54 ± 3.09 years. All participants were recruited from universities in Nanchang, Jiangxi Province, China. In terms of geographical distribution within the sample, 63.1% of the participants belonged to urban regions, 36.7% belonged to rural regions, and 0.2% did not report their place of origin. Participants were not screened for chronic comorbidities prior to survey completion. This represents a significant limitation for a respiratory infection study, as conditions such as asthma (prevalence ~5–9% in college students), exercise-induced bronchoconstriction, and allergic rhinitis can independently mimic COVID-19 respiratory symptoms and restrict physical activity levels. These unmeasured confounders threaten the internal validity of our findings regarding the relationship between COVID-19 experience and physical activity. While the prevalence of chronic diseases is relatively low in young adults (mean age 19.54 ± 3.09 years), respiratory comorbidities are indeed prevalent in this age group and may substantially influence both symptom reporting and physical activity patterns. Participants were recruited from the general university student population across various academic disciplines. Sensitivity analysis excluding participants reporting high-performance athletic participation (e.g., varsity team members, competitive athletes) yielded results consistent with the main findings (*r* = −0.192, *p* < 0.01 for males; r = −0.048, *p* > 0.05 for females), suggesting that elite athlete status did not substantially influence the observed associations our sample is presumed to predominantly represent recreational exercisers and sedentary individuals.

### 2.3 Measurement tools

#### 2.3.1 Physical Activity Rating Scale-3

The Physical Activity Rating Scale-3 (PARS-3), revised by Liang (12), was used to assess the level of physical activity. The scale consists of three indicators, namely, intensity, duration, and frequency of physical activity, each represented by a single question. The intensity of physical activity is categorized into five levels as follows: low, low-moderate, moderate, moderate-high, and high. The duration of physical activity is categorized into five levels as follows: <10 min, 11–20 min, 21–30 min, 31–59 min, and ≥60 min. The frequency of physical activity is categorized into five levels as follows: <1 time per month, 2–3 times per month, 1–2 times per week, 3–5 times per week, and ≥7 times per week. Based on a Likert scale, intensity and frequency were graded from 1 to 5, each scored from 1 to 5, respectively, and duration was graded from 1 to 5, each scored from 0 to 4, respectively. The total physical activity score was calculated using the original formula proposed by Liang (12): score = intensity score × (duration score – 1) × frequency score, with a possible range of 0–100, where as the minimum score was 0 points. The level of physical activity was classified as follows: fluctuating, a total score of ≤19; medium, a total score of 20–42; and high, a total score of ≥43. The internal consistency reliability (*α*) of the scale was 0.82, whereas the Kaiser–Meyer–Olkin (KMO) value was 0.717.

#### 2.3.2 Wisconsin Upper Respiratory Symptom Survey-21

The Wisconsin Upper Respiratory Symptom Survey-21 (WURSS-21), developed and validated by Barrett et al. (13) for assessing common cold and influenza-like illness severity, was employed in this study to capture upper respiratory and functional symptoms. We explicitly acknowledge that the WURSS-21 was not developed or validated specifically for COVID-19 severity assessment. Its use in this study is supported by symptomatic overlap between mild-to-moderate COVID-19 and common URTIs/influenza-like illnesses, with shared symptoms (e.g., nasal congestion, sore throat, cough, fatigue, headache, body aches) aligning with WURSS-21 domains (13–17). However, we explicitly acknowledge the following critical limitations: (1) COVID-19 frequently involves lower respiratory symptoms (dyspnea, pneumonia) that are not captured by WURSS-21; (2) loss of taste/smell—a cardinal symptom of COVID-19—is not assessed by this instrument; and (3) the scale’s performance for discriminating COVID-19 severity has not been established. No validated severity assessment tool specific to COVID-19 was available at the time of this study. Although COVID-19 can involve the lower respiratory tract in more severe cases (e.g., pneumonia or acute respiratory distress), its milder presentations frequently manifest with upper respiratory and systemic symptoms compatible with WURSS-21 assessment (15, 18). Therefore, relevant sections of the WURSS-21 were used in this study to capture symptom severity and quality-of-life impact post-recovery. The instrument comprises three domains (nasal symptoms, throat symptoms, and activity/function) across 16 items, each scored on a 7-point Likert scale (0 = not sick; 1 = very mild; 3 = mild; 5 = moderate; 7 = severe). Domain scores were summed accordingly. In the present sample, subscale KMO values were 0.888 (nasal), 0.734 (throat), and 0.893 (activity/function); overall KMO was 0.947, with Cronbach’s *α* = 0.944. The WURSS-21 total scores were divided into four equal quartiles (Q1: Quartile 1, Q2: Quartile 2, Q3: Quartile 3, Q4: Quartile 4) for analytical purposes. We acknowledge that these severity subgroups are based on mathematical quartiles rather than clinically validated cut-off points, which may limit the clinical interpretability of these specific categorical boundaries.

### 2.4 Data analysis

Statistical analysis was performed using the SPSS (version 27.00) and Excel 2021 software. Spearman correlation, canonical correlation, regression, and covariance analyses were performed to examine the relationships between variables. A *p*-value of <0.05 indicated statistically significant differences. A *priori* power analysis indicated that our sample size (*N* = 1,633) provided >99% power to detect small correlations (*r* = 0.07) at *α* = 0.05. To control for family-wise error rate across multiple comparisons, we applied the Bonferroni correction. For the 10 correlation coefficients in Table 1, the adjusted significance threshold was *α* = 0.005 (0.05/10).

**Table 1:** Correlation analysis of the experience of COVID-19 and the level of physical activity after recovery in college students.

Variables	*M* ± SD	1	2	3	4	5
1. Nasal	32.65 ± 14.45	—				
2. Throat	14.33 ± 7.12	0.825^**^	—			
3. Activity and function	21.23 ± 11.04	0.801^**^	0.628^**^	—		
4. Infection experience	59.96 ± 27.35	0.964^**^	0.830^**^	0.917^**^	—	
5. Level of physical activity	18.20 ± 22.89	−0.120^**^	−0.066^**^	−0.114^**^	−0.230^**^	—

*p* < < 0.01 (Bonferroni-corrected threshold: *p* < < 0.005 for 10 comparisons).

^a^ Variable 4 = composite of Variables 1–3.

^b^ Variable 5 = composite of intensity, duration, and frequency.

** indicates *p* < 0.01.

## 3 Results

### 3.1 Relationship between COVID-19 infection experience and the level of physical activity among college students after recovery

Correlation analysis was performed to examine the relationship between COVID-19 and the level of physical activity among college students after recovery. The results indicated that variables related to nasal symptoms, throat symptoms, activity and function, and overall symptoms were significantly correlated with the level of physical activity after recovery from COVID-19 (*p* < 0.01). Canonical correlation analysis (CCA) revealed a significant multivariate relationship between the symptom variables and physical activity variables, *Wilks’ λ* = 0.980, *χ*^2^(6) = 32.390, *p* < 0.001. Canonical correlation analysis (CCA) revealed a first canonical correlation of 0.118 [Wilks’ λ = 0.980, *χ*^2^(6) = 32.390, *p* < 0.001]. However, redundancy analysis revealed negligible explanatory power (redundancy indices < 1%), indicating that the canonical variates explain less than 1% of the variance in the opposing variable set. Despite statistical significance—driven by the large sample size—the canonical correlation of 0.118 represents a trivial effect (*r*^2^ = 0.014) with no practical significance. Standardized coefficients are reported for descriptive purposes but should not be interpreted as indicating meaningful predictor importance given the negligible overall explanatory power, indicating that the canonical correlation explains minimal variance in the dependent variables. Detailed information is shown in Table 1.

### 3.2 Key indicators of COVID-19 influencing the level of physical activity in college students after recovery

**Figure 1:**
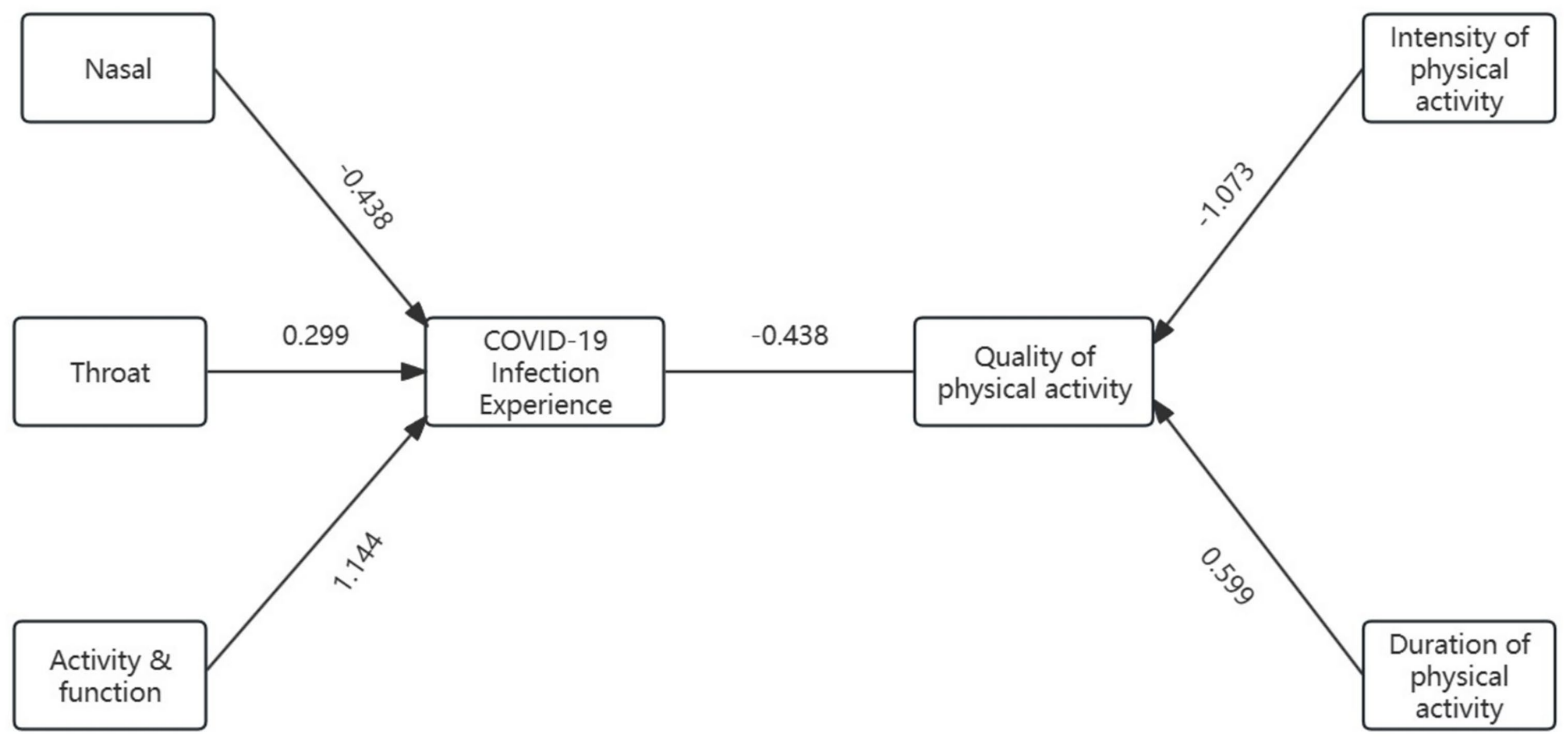
The canonical correlation analysis between the experience of COVID-19 and the level physical activity after recovery in college students.

Covariance analysis showed that the first three variables of COVID-19 explained 69.325% of the total variance and one variable of the level of physical activity explained 59.943% of the total variance. Correlation analysis revealed three pairs of correlation models; among which, the first pair was selected: U1 = −0.438X (nasal symptoms) + 0.299X (pharyngeal symptoms) + 1.144X (activity and function) and V1 = −1.073Y (intensity of physical activity) + 0.599Y (duration of physical activity) (*p* < 0.001). The experience of COVID-19 showed a statistical association with the level of physical activity (Figure 1).

### 3.3 Sex differences in the relationship between experience of COVID-19 and the level of physical activity in college students after recovery

Sex was included as a covariate in covariance analysis. The results showed that sex had a significant effect (*p* < 0.05) on the relationship between COVID-19 and the level of physical activity among college students after recovery. After recovery, male college students demonstrated a higher average level of physical activity compared with female students [*M* = 22.86 ± 25.91 vs. *M* = 13.90 ± 18.70; small effect size (*r* = −0.230, *r*^2^ = 0.015, explaining 1.5% of variance)]. However, a negative correlation was observed between physical activity level and infection experience severity among male students (*r* = −0.183, *p* < 0.001), whereas that of female students showed no statistically significant trend (*r* = −0.045, *p* > 0.05). Furthermore, among male students, significant negative Spearman correlations were observed between COVID-19 infection experiences and all three dimensions of physical activity: intensity (*r* = −0.129, *p* < 0.01), duration (*r* = −0.080, *p* < 0.05), and frequency (*r* = −0.106, *p* < 0.01). Among female students, infection experiences were negatively correlated with physical activity intensity (*r* = −0.081, *p* < 0.05), but not significantly correlated with duration (*r* = 0.061) or frequency (*r* = 0.076). Overall, male students’ infection experiences were significantly negatively correlated with all three indicators of physical activity level (*p* < 0.05). In contrast, female students exhibited a significant negative correlation only with physical activity intensity (*p* < 0.05), while correlations with the other two indicators were positive though not statistically significant (see Tables 2, 3).

**Table 2:** The status of public sports venues after COVID-19 in China.

Number	Time	Place	Status
1	2023.2.6	Tongxu County, Henan	The baffle has always existed around People’s Stadium of Tongxu County at the end of 2022, and the residents were still unable to enter.
2	2022.12.13	Toudaowan Community, Xinjiang	The community administrator locked the indoor basketball gym, and refused anyone enter with various reasons after COVID-19.
3	2023.7.5	Wuxi, Zhejiang	Although half a year passed after China manage COVID-19 with measures against Class B infectious diseases, the stadium in Wuxi Sports School has not yet opened to the public.
4	2023.4.4	Shanghai	The stadiums in primary and secondary schools in Shanghai began to open to the public until April, 2023.
5	2023.9.10	Ji’nan, Shandong	The stadiums in primary and secondary schools in Ji’nan began to open to the public until September, 2023, and the citizens can go to do exercise during holidays, weekend and spare time.

The information collected from online sources.

**Table 3:** Canonical correlation analysis of COVID-19 symptoms and physical activity dimensions.

Parameter	Value
Wilks’ *λ*	0.98
*χ*^2^ (df = 6)	32.39
*p*-value	<< 0.001
First canonical correlation	0.118
Redundancy index (U\|V)	<< 1%
Redundancy index (V\|U)	<< 1%

### 3.4 Potential of COVID-19 in predicting the level of physical activity in college students after recovery

For regression analysis, the level of physical activity after recovery from COVID-19 among college students was selected as the dependent variable, which was denoted as Y, where as the severity of COVID-19 experience was selected as the independent variable, which was denoted as X. The regression equation for determining the relationship between the severity of COVID-19 experience and the level of physical activity was Y1 = −3.20×1 + 27.181, *F*(1, 1,631) = 25.348, *p* < 0.001. The effect size was small (*R*^2^ = 0.015, Adjusted *R*^2^ = 0.015), indicating that COVID-19 experience severity explains only 1.5% of the variance in physical activity levels. Given this minimal explanatory power, the statistical significance is likely driven by the large sample size (*N* = 1,633) rather than a substantively meaningful association. The unstandardized regression coefficient [*B* = −3.200, 95% CI (−4.447, −1.953), *β* = −0.124] indicated that for each one-level increase in COVID-19 infection severity, the level of physical activity decreased by 3.20 points (*N* = 1,633) which indicated that the level of physical activity decreased with an increase in the severity of COVID-19 experience. In particular, the level of physical activity decreased by 3.20 points for each level of increase in the severity of COVID-19 experience. The regression equations for determining the relationship between the severity of COVID-19 experience and the level of physical activity among male and female college students were: For male students (Y2): Y2 = −5.98×2 + 39.96, *F*(1, 773) = 28.471, *p* < 0.001, *R*^2^ = 0.035, Adjusted *R*^2^ = 0.034. This represents the largest effect size in our analyses, yet still explains only 3.5% of the variance, indicating limited practical significance despite statistical significance. The unstandardized regression coefficient [*B* = −5.980, 95% CI (−7.179, −4.781), *β* = −0.189] indicated that for each one-level increase in COVID-19 infection severity, the level of physical activity decreased by 5.98 points among male students. For female students (Y3): Y3 = −2.79×3 + 22.76, *F*(1, 856) = 6.741, *p* = 0.010, *R*^2^ = 0.008, Adjusted *R*^2^ = 0.007. This negligible effect size (explaining less than 1% of variance) suggests no practically meaningful association in this subgroup. The unstandardized regression coefficient [*B* = −2.790, 95% CI (−4.902, −0.678), *β* = −0.089] indicated that for each one-level increase in COVID-19 infection severity, the level of physical activity decreased by 2.79 points among female students.

## 4 Discussion

Interpretation Caveats: It is essential to emphasize that the cross-sectional design of this study precludes any causal inference. The observed correlations (|*r*| = 0.045–0.230) represent weak associations that explain 0.2–5.3% of the variance in physical activity levels. The statistical significance of these trivial effects is attributable to the large sample size (*N* = 1,633), which provides >99% power to detect correlations as small as *r* = 0.07. These findings should not be interpreted as indicating meaningful, clinically relevant, or causally interpretable relationships between COVID-19 infection experience and physical activity behavior.

In their study on COVID-19, Sallis et al. (19) demonstrated that individuals who engaged in regular physical exercise had lower rates of hospitalization, ICU admission, and mortality compared to those who were consistently inactive (19, 20). Moreover, the pandemic coincided with heightened public awareness of the importance of physical and mental health (21), which may be associated with increased participation in physical activity once restrictions are lifted or relaxed (22). Previous studies have also encouraged a return to sports (23). However, the present study revealed a statistically significant negative correlation between university students’ infection experience and their current physical activity level (*r* = −0.230, *p* < 0.01), with a small effect size (*r*^2^ = 0.015, explaining 1.5% of variance). It is critical to emphasize that this effect size indicates minimal practical significance: 98.5% of the variance in physical activity levels remains unexplained by COVID-19 experience. The statistical significance is attributable to the large sample size (*N* = 1,633), which provides >99% power to detect trivial effects (*r* = 0.07). Consequently, these findings should not be interpreted as indicating a meaningful or clinically relevant association. The regression equation (Y1 = −3.20×1 + 27.181) similarly reflects a weak statistical relationship with negligible predictive utility (*R*^2^ = 0.015).

While the statistical association is weak, we note the following contextual factors that may coincide with the observed pattern: (1) higher severity of COVID-19 experience coincided with greater reported suffering after infection, and concerns about exercise increasing infection risk were more frequently reported among those with severe experiences (24, 25). Several countries worldwide banned public gatherings during the pandemic to avoid re-infection, a period that coincided with lower physical activity levels. (2) A total of 93% of individuals reportedly considered “physical health” the most important thing in life in the post-pandemic period (26, 27). However, owing to the failure of public sports venues or services to resume in a timely manner, the motivation reported during the pandemic was not associated with higher real-life behavior, and instead, was associated with lower levels of physical activity. Therefore, after disease outbreaks, sports, health, and medical departments may consider increasing awareness regarding exercise and helping people understand the scientific relationship between disease severity and physical activity. This approach may be hypothesized to coincide with reduced fear of re-infection and potentially higher physical activity participation, though causal effects cannot be established from this study. Higher physical activity levels are associated with improved immunity and resistance in previous research.

This study showed that the effects of COVID-19 on the level of physical activity in the post-recovery phase were different between male and female college students. Although the average scores of physical activity level were higher among male students than among female students, a negative correlation was observed between physical activity level and COVID-19 experience severity among male students, On the contrary, no consistent correlation pattern was observed between physical activity level and COVID-19 experience severity among female students. These differences may be hypothesized to relate to the following factors: (1) women may have more nerve fiber receptors than men, potentially resulting in a stronger and more sensitive response to pain (28). Therefore, female students reported higher severity of COVID-19 experience than male students. (2) Some men reportedly did not pay much attention to the pandemic (29); for example, some women have reported that “My boyfriend did not care about the outbreak at all and said that I was over-anxious” (30). This behavior may have been associated with male students’ judgment of the infection experience. (3) Owing to differences in immune systems, sex hormones, and other factors, COVID-19 affected men more severely than women. As a result, men could not participate in physical activity in time to rest ore their physical condition (31–33); (4) COVID-19 infection can lead to muscle damage, such as reduced muscle mass (34). Men and women often report different goals for physical exercise: men typically focus on strength and endurance, whereas women are more likely to prioritize health, weight management, and body shape (35). This hypothesizes that women’s heightened awareness of bodily signals post-infection, coupled with concerns about weight management, may be associated with closer attention to recovery. Consequently, this heightened awareness could foster a more positive bodily perception—such as perceiving faster healing and rehabilitation. This perception could enable them to return to physical activity earlier and enhance their motivation to engage in exercise. In contrast, men may be differentially affected in this context: they might underestimate their physical discomfort relative to the actual extent of impairment and, due to muscle damage, maybe temporarily unable to participate in physical activities therefore, they may require a longer recovery period and develop more persistent memories of the infection, which in turn may be associated with attenuated physical behavior. The following measures may be considered in relation to physical activity participation after a disease outbreak: (1) Public sports service policies may be reviewed in accordance with disease control policies, and the reopening of public sports venues closed during disease outbreaks may help address post-outbreak fitness needs. (2) Communities may consider monitoring changes in residents’ sports participation after disease outbreaks. Efforts to address fitness needs through community sports events, venue access, fitness guidance, and equipment distribution may be beneficial. (3) Given that social gatherings are restricted and fitness centers are closed during disease outbreaks, family members family members may encourage and support each other to maintain a home exercise regime through online training sessions and participate in online sports events. (4) Universities may consider monitoring changes in college students’ sports participation in the post-outbreak period and developing programs that address post-outbreak fitness needs. Additionally, society as a whole should organize fitness activities and provide fitness services at the national level, creating a conducive environment for improving physical well-being.

This study showed that male college students reported lower physical activity levels associated with COVID-19 experience compared to female students. In particular, fourth-year students showed a positive correlation between COVID-19 experience severity and physical activity levels, though this finding should be interpreted cautiously given the small effect size and cross-sectional design. Studies have shown that men are more susceptible than women to COVID-19 (36) and experience more severe symptoms after infection, with delayed physical recovery (37–39). Fourth-year college students reported higher perceived stress levels (related to employment and graduation), which may coincide with different physical activity patterns (40). A positive correlation was observed between infection experience severity and physical activity levels among this group. Physical activity is associated with physical well-being, stress management, and physical fitness in previous research, though causal pathways cannot be inferred from the present cross-sectional data. Post-outbreak public sports services may consider demographic differences and group-oriented initiatives. Activities such as yoga, tai chi, and other outdoor exercises may be associated with stress management and fitness maintenance in previous research. Targeted public sports services may support physical and mental health and fitness participation, contributing to the development of mass sports in the post-outbreak period, though causal efficacy cannot be established.

Our findings should be interpreted within the context of a general college student population. Elite athletes, who constitute a small proportion of university students, may demonstrate distinct patterns of physical activity recovery following COVID-19 due to their higher baseline fitness levels, structured training environments, and potentially different physiological responses to viral infection (41, 42). The absence of elite athlete status assessment limits our ability to generalize findings to high-performance athletic populations.

## 5 Conclusion

This study showed that the experience of COVID-19 was significantly negatively correlated with the level of physical activity among college students in the post-recovery phase. In particular, male students reported lower physical activity levels than female students, which may reflect different baseline patterns or reporting tendencies. Furthermore, the level of physical activity was positively associated with the experience of COVID-19 among fourth-year students, which may be related to high levels of stress regarding graduation and employment. Potential strategies associated with physical activity participation after disease outbreaks include: (1) strengthening public sports services and addressing psychological barriers; (2) organizing mass sports events to raise awareness of physical fitness; and (3) considering population differences in the association between disease experience and physical fitness when developing targeted measures.

## 6 Research limitations

This study has several important limitations. First, the cross-sectional design precludes causal inference; we can only report associations, not effects or impacts. Second, we lack pre-infection physical activity data, making it impossible to determine whether observed lower levels reflect new changes or pre-existing low activity levels. Third, multiple confounding variables were not measured or controlled, including pre-existing health conditions, long COVID symptoms, socioeconomic status, sport participation type, and baseline exercise habits. Fourth, the sample consisted of convenience-sampled university students from a single city (Nanchang, Jiangxi Province), limiting generalizability to other populations (e.g., different age groups, geographic regions, education levels). China exhibits substantial regional variation in COVID-19 infection rates, public health policies, and physical activity infrastructure. Findings from this predominantly urban, central Chinese sample may not extend to students in other provinces, rural areas, or regions with different pandemic experiences. Fifth, the use of ‘d’ as an effect size measure in some analyses is non-standard and potentially misleading. Sixth, we did not collect data on pre-existing chronic comorbidities (e.g., hypertension, diabetes, respiratory disorders, or cardiovascular diseases) that could potentially influence physical activity levels and COVID-19 recovery trajectories. While the prevalence of such conditions is relatively low in young adult populations (43), we must explicitly acknowledge that respiratory conditions such as asthma, exercise-induced bronchoconstriction, and allergic rhinitis are indeed prevalent among college students (reported prevalence of 5–9% in this age group) and represent a significant limitation of this study. These conditions can mimic COVID-19 respiratory symptoms and independently restrict physical activity levels, thereby threatening the internal validity of our findings regarding the relationship between COVID-19 severity and physical activity. Future studies should specifically screen for and control these respiratory comorbidities. Seventh, we did not assess or control for elite athlete status or high-level sports participation. Elite athletes possess distinct physiological profiles and may exhibit different COVID-19 recovery patterns and physical activity behaviors compared to recreational exercisers or sedentary individuals while the proportion of elite athletes in our general university sample is likely low, future studies should consider stratifying analyses by athletic status or excluding high-performance athletes to ensure homogeneity future studies should consider including these variables as potential confounders or exclusion criteria and consistently report conventional metrics such as R^2^ and *β* coefficients. Eighth, the classification of COVID-19 severity was based on quartile distribution of WURSS-21 scores rather than clinically validated severity thresholds. This approach, while enabling statistical comparison across severity levels, lacks clinical validation and may not accurately reflect medically meaningful differences in disease severity. Future studies should consider using clinically established severity criteria or validated severity scoring systems for COVID-19. Ninth, the effect sizes observed in this study (*R*^2^ = 0.015–0.035) are minimal, explaining less than 4% of the variance in physical activity levels even in the subgroup with the strongest association (male students). The large sample size (*N* = 1,633) ensures statistical significance for these trivial effects, but the findings have limited practical or clinical relevance. Readers should not interpret statistical significance as indicative of meaningful associations. Tenth, both the exposure (COVID-19 symptoms via WURSS-21) and outcome (physical activity via PARS-3) were assessed through self-report, introducing potential self-report bias and common method variance. The lack of objective measures (e.g., accelerometry for physical activity; serological or PCR confirmation for COVID-19 infection) limits the validity of our measurements. Eleventh, the six-month retrospective recall period for both infection symptoms and physical activity introduces recall bias. Participants may have difficulty accurately remembering symptom severity and activity patterns over extended periods, and the temporal sequence between infection experience and activity levels cannot be established. Twelfth, the WURSS-21 was not developed or validated for COVID-19 severity assessment. This instrument does not capture lower respiratory symptoms (dyspnea, pneumonia) or chemosensory dysfunction (anosmia/ageusia) characteristic of COVID-19, and its performance for discriminating COVID-19 severity has not been established.

## Data Availability

The data analyzed in this study is subject to the following licenses/restrictions: (1) a confirmed history of COVID-19 infection as indicated by a total score of >0 on the Wisconsin Upper Respiratory Symptom Survey (WURSS-21) scale; (2) university students; and (3) completion of the questionnaire. Requests to access these datasets should be directed to ZG, 3581619939@qq.com.

## Footnotes

1
https://www.wjx.cn/vm/eqYrTp6.aspx

## References

[1] Jamison DT Gelband H Horton S Jha P Laxminarayan R Mock CN . In: editors, editor. Disease Control Priorities: Improving Health and Reducing Poverty, 3rd Edn. Washington (DC): The International Bank for Reconstruction and Development/The World Bank (2017) 30212058

[2] Scoil an Leighis treidliachta UCD. (2020). From the Archives: The Spanish Flu Pandemic, 1918–1919[EB/OL]. Available online at: https://www.ucd.ie/vetmed/about/alumni/alumninewsletter-may2020/fromthearchivesthespanishflupandemic1918-1919 (Accessed December 20, 2024).

[3] Lau JT Yang X Tsui HY Kim JH. Impacts of SARS on health-seeking behaviors in general population in Hong Kong. Prev Med. (2005) 41:454–62. doi: 10.1016/j.ypmed.2004.11.023, 15917041 PMC7119319

[4] Karlberg J Chong DS Lai WY. Do men have a higher case fatality rate of severe acute respiratory syndrome than women do? Am J Epidemiol. (2004) 159:229–31. doi: 10.1093/aje/kwh056, 14742282 PMC7110237

[5] Lazarus RS. Progress on a cognitive-motivational-relational theory of emotion. Am Psychol. (1991) 461:819–34. doi: 10.1037//0003-066x.46.8.819 1928936

[6] Coull A Pugh G. Maintaining physical activity following myocardial infarction: a qualitative study. BMC Cardiovasc Disord. (2021) 21:105. doi: 10.1186/s12872-021-01898-7, 33602122 PMC7893716

[7] Schneider KL Andrews CA Hovey K Andrews C Hovey KM Seguin RA . Change in physical activity after a diabetes diagnosis: opportunity for intervention. Med Sci Sports Exerc. (2014) 46:84–91. doi: 10.1249/MSS.0b013e3182a33010, 23860414 PMC4028702

[8] Mo L. (2003). What else should the sports sector do in the wake of SARS. Available online at: http://sports.sina.com.cn/s/2003-06-21/1710471926.shtml, (Accessed October 24, 2023).

[9] Li Q. (2022). The epidemic is improving and the public is enthusiastic about sports. Available online at: https://www.mm111.net/2022/07/24/991243586.html (Accessed November 26, 2023).

[10] Cleveland Clinic. (2021). Lessons from the pandemic: survey reveals vulnerabilities in Americans’ commitment to preventive health. Available online at: https://newsroom.clevelandclinic.org/2021/05/11/lessons-from-the-pandemic-survey-reveals-vulnerabilities-in-americans-commitment-to-preventive-health/ (Accessed June 12, 2023).

[11] Park AH Zhong S Yang H Jeong J Lee C. Impact of COVID-19 on physical activity: a rapid review. J Glob Health. (2022) 12:05003. doi: 10.7189/jogh.12.05003, 35493780 PMC8979477

[12] Liang DQ. Stress levels and their relationship with physical activity among college students. Chin Ment Health J. (1994) 8:5–6.

[13] Barrett B Locken K Maberry R Schwamman J Brown R Bobula J . The Wisconsin upper respiratory symptom survey (WURSS): a new research instrument for assessing the common cold. J Fam Pract. (2002) 518:265–73. 11978238

[14] Department of Family Medicine and Community Health (n.d.) Wisconsin upper respiratory symptom survey (WURSS). Official website. Available online at: https://www.fammed.wisc.edu/wurss/ (Accessed December 20, 2024).

[15] Astuti I. Severe acute respiratory syndrome coronavirus 2 (SARS-CoV-2): an overview of viral structure and host response. Diabetes Metab Syndr. (2020) 14:407–12. doi: 10.1016/j.dsx.2020.04.020, 32335367 PMC7165108

[16] Chauhan S. Comprehensive review of coronavirus disease 2019 (COVID-19). Biom J. (2020) 43:334–40. doi: 10.1016/j.bj.2020.05.023, 32788071 PMC7263230

[17] Pal M Berhanu G Desalegn C Kandi V. Severe acute respiratory syndrome Coronavirus-2 (SARS-CoV-2): an update. Cureus. (2020) 12:e7423. doi: 10.7759/cureus.7423, 32337143 PMC7182166

[18] Compton J. (2023). How the pandemic has changed working out at the gym for the better. Available online at: https://www.today.com/health/diet-fitness/pandemic-changed-working-out-gym-rcna28364 (Accessed September 6, 2023).

[19] Sallis R Young DR Tartof SY Sallis JF Sall J Li Q . Physical inactivity is associated with a higher risk for severe COVID-19 outcomes: a study in 48 440 adult patients. Br J Sports Med. (2021) 55:1099–105. doi: 10.1136/bjsports-2021-104080, 33849909

[20] World Health Organization (WHO). (2020). Coronavirus disease (COVID-19): similarities and differences between COVID-19 and influenza. Available online at: https://www.who.int/news-room/questions-and-answers/item/coronavirus-disease-covid-19-similarities-and-differences-with-influenza (Accessed October 14, 2024).

[21] Lin R Hu X Guo L Huang J. The health benefit of physical exercise on COVID-19 pandemic: evidence from mainland China. PLoS One. (2022) 17:e0275425. doi: 10.1371/journal.pone.0275425, 36223368 PMC9555623

[22] Reynolds G. (2023). How we exercise is different now—the pandemic changed everything. Available online at: https://www.irishtimes.com/life-and-style/health-family/fitness/how-we-exercise-is-different-now-the-pandemic-changed-everything-1.4376559 (Accessed August 20, 2023).

[23] Andrade A Dominski FH Pereira ML de Liz CM Buonanno G. Infection risk in gyms during physical exercise. Environ Sci Pollut Res Int. (2018) 25:19675–86. doi: 10.1007/s11356-018-1822-8, 29736645

[24] Hughes DC Orchard JW Partridge EM La Gerche A Broderick C. Return to exercise post-COVID-19 infection: a pragmatic approach in mid-2022. J Sci Med Sport. (2022) 25:544–7. doi: 10.1016/j.jsams.2022.06.001, 35725689 PMC9170595

[25] Chen Z. Zhou L. T.. (2021). Post-pandemic era 93% of people say "good health" is Most important. Available online at: http://health.ce.cn/news/202111/16/t20211116_7331878.shtml (Accessed August 12, 2023).

[26] Cleveland Clinic. (2024). Is it OK to exercise with COVID-19? [EB/OL]. Available online at: https://health.clevelandclinic.org/exercise-with-covid (Accessed December 20, 2024).

[27] UNICEF for every child. (2021). During crisis we realized, health is the most important thing [EB/OL]. Available online at: https://www.unicef.org/eu/stories/during-crisis-we-realized-health-most-important-thing (Accessed December 20, 2024).

[28] Peng X Ma SH Li XM. Gender differences in cognitive styles of college students. Chin J Health Psychol. (2006) 14:299–301.

[29] Psychology Today. (2021). Why women and men have different fears about Covid-19 [EB/OL]. Available online at: https://health.clevelandclinic.org/exercise-with-covid (Accessed December 20, 2024).

[30] Hu X S. (2022). My boyfriend doesn't care about the epidemic at all and says I'm over-anxious [EB/OL]. Available online at: https://baijiahao.baidu.com/s?id=1731227872185401521&wfr=spider&for=pc (Accessed November 28, 2023).

[31] Montopoli M Zumerle S Vettor R Rugge M Zorzi M Catapano CV . Androgen-deprivation therapies for prostate cancer and risk of infection by SARS-CoV-2: a population-based study (n = 4532). Ann Oncol. (2020) 31:1040–5. doi: 10.1016/j.annonc.2020.04.479, 32387456 PMC7202813

[32] Klein SL Marriott I Fish EN. Sex-based differences in immune function and responses to vaccination. Trans R Soc Trop Med Hyg. (2015) 109:9–15. doi: 10.1093/trstmh/tru167, 25573105 PMC4447843

[33] Schroeder M Tuku B Jarczak D Nierhaus A Bai T Jacobsen H . The majority of male patients with COVID-19 present low testosterone levels on admission to intensive care in Hamburg, Germany: a retrospective cohort study. medRxiv. (2020). doi: 10.1101/2020.05.07.20073817

[34] McCray PBJ Pewe L Wohlford-Lenane C Hickey M Manzel L Shi L . Lethal infection of K18-hACE2 mice infected with severe acute respiratory syndrome coronavirus. J Virol. (2007) 81:813–21. doi: 10.1128/JVI.02012-06, 17079315 PMC1797474

[35] Elijah GR Eric DKN. Motivational gender differences in sport and exercise participation among university sport science students. J Phys Educ Sport. (2012) 12:180–7.

[36] Araneo BA Dowell T Diegel M Daynes RA. Dihydrotestosterone exerts a depressive influence on the production of interleukin-4 (IL-4), IL-5, and gamma-interferon, but not IL-2 by activated murine T cells. Blood. (1991) 78:688–99. doi: 10.1182/blood.V78.3.688.688, 1830499

[37] Lipsky MS Hung M. Men and COVID-19: a pathophysiologic review. Am J Mens Health. (2020) 14:813–821. doi: 10.1177/1557988320954021, 32936693 PMC7495118

[38] Barrat F Lesourd B Boulouis HJ Thibault D Vincent-Naulleau S Gjata B . Sex and parity modulate cytokine production during murine ageing. Clin Exp Immunol. (1997) 109:562–8. doi: 10.1046/j.1365-2249.1997.4851387.x, 9328137 PMC1904767

[39] Roberts CW Walker W Alexander J. Sex-associated hormones and immunity to protozoan parasites. Clin Microbiol Rev. (2001) 14:476–88. doi: 10.1128/CMR.14.3.476-488.2001, 11432809 PMC88985

[40] Yao LC. The Relationship Between Psychological Resilience and Post-Stress Growth of College Students: Chain Mediating of Perception Stress and Positive Coping Styles. Shenyang, China: Shenyang Normal University (2022).

[41] Schumacher YO Tabben M Hassoun K Al Marwani A Al Hussein I Coyle P . Resuming professional football (soccer) during the COVID-19 pandemic: experiences and concerns of 729 players from the German Bundesliga. Br J Sports Med. (2021) 55:793–8. doi: 10.1136/bjsports-2020-103724 PMC7886664 33589470

[42] Sarto F Impellizzeri FM Spörri J Porcelli S Olmo J Requena B . Impact of potential physiological changes due to COVID-19 home confinement on athlete health protection in elite sports: a call for awareness in sports programming. Sports Med. (2020) 50:1417–9. doi: 10.1007/s40279-020-01297-6, 32468329 PMC7254973

[43] Liu M Cao L Wang L Zhou X Qiu J Liu S . Prevalence and risk factors of hypertension among Chinese college students: a systematic review and meta-analysis. J Hypertens. (2021) 39:1261–9. doi: 10.1097/HJH.0000000000002789

